# Tumor-associated T cell receptor repertoires in low- and high-grade gliomas

**DOI:** 10.1186/2051-1426-1-S1-P143

**Published:** 2013-11-07

**Authors:** Jennifer S Sims, Boris Grinshpun, Benjamin I Amendolara, Yufeng Shen, Peter D Canoll, Peter A Sims, Jeffrey N Bruce

**Affiliations:** 1 Dept of Neurological Surgery, Columbia University, New York, NY, USA; 2 Dept of Pathology and Cell Biology, Columbia University, New York, NY, USA; 3 Dept of Systems Biology, Columbia University, New York, NY, USA; 4 Dept of Biomedical Informatics, Columbia University, New York, NY, USA; 5 Dept of Biochemistry & Molecular Biophysics, Columbia University, New York, NY, USA

## 

Glioblastoma (GBM) remains prognostically dismal, with care centered on resection, motivating research into novel therapies. Although inducing anti-tumor immunity remains an attractive target for therapeutic and preventative intervention, the interplay between evolving dysregulation of the glioma microenvironment and T cell inefficacy remains poorly understood. In our murine model of proneural glioma, retroviral delivery of PDGF and cre-mediated knockout of PTEN in glial progenitors of adult C57BL/6 gives rise to slow-growing tumors, which were harvested at early- mid- and late-stage progression timepoints following induction, along with peripheral blood. From human patients, tissue from low- and high-grade glioma resections and corresponding peripheral lymphocytes were cryofrozen during surgery at New York Presbyterian-CUMC. For both species, we employed a commercially available primer set (iRepertoire) for nested PCR of the complementarity-determining region 3 (CDR3) of the TCR-alpha and TCR-beta chains from the T cell RNA, followed by next-generation sequencing on an Illumina MiSeq. We developed a computational pipeline for mapping TCR cassettes, in silico translation, pairwise analysis of tissue/periphery per subject, and error analysis. In the murine model, we observe that at late-stage, the intratumoral TCR repertoire diverges significantly from the peripheral, including dramatic expansion of single tumor-associated CDR3s, while the peripheral repertoire itself diverges from those of healthy mice. In both human patients and mice, we observed tumor-associated CDR3s, disproportionately abundant in tumor tissue compared to the corresponding peripheral blood, at both the amino acid and nucleotide level. In human samples we observed tumor-specific TCR expansions that were associated with particular functional subsets (CD8+, CD4+, Treg, NKT). Sequence-level study of the TCR repertoire promises new insight into the scope of glioma immunosuppression, especially systemic effects which remain elusive and the origins of intratumoral suppressive populations, and holds the potential for immunotherapeutic interventions, non-invasive diagnostics, and direct assessment of global responses to immunotherapy.

